# Evaluation of the protective effects of *Spirulina platensis* against cyclophosphamide-induced genotoxicity

**DOI:** 10.1186/s42826-025-00242-w

**Published:** 2025-04-22

**Authors:** Kouamé Ephrem Zikpi, Aku Enam Motto, Kokou Atchou, Kounouho R. Adounkpe Kougblenou, Povi Lawson-Evi, Kwashie Eklu-Gadegbeku

**Affiliations:** 1 Laboratory of Physiology/ Pharmacology, Research Unit of Pathophysiology, Bioactive Substances and Safety (PSBI), Faculty of Sciences, University of Lomé, Lomé, 01 BP1515 Togo; 2 Regional Institute for Development and Health (IREDESA), Ex CREDESA (Centre Régional pour le Développement et la Santé), Pahou, Cotonou, 01 BP1822 Benin

**Keywords:** *Spirulina platensis*, Cyclophosphamide, Anti-genotoxicity, In vivo, Antioxidant

## Abstract

**Background:**

Damage to normal cells is the most common limitation of cancer chemotherapy. Cyclophosphamide, one of the most widely used anticancer drugs due to its cytotoxicity, can bind to deoxyribonucleic acid (DNA), causing chromosomal breaks, micronuclei, and cell death. The use of natural sources helps to prevent this damage, and *Spirulina platensis* is highly appreciated for its numerous bioactive compounds. This study aimed to investigate the antigenotoxic effects of *Spirulina platensis* powder (PoSP) on mouse bone marrow cells in vivo *via a micronucleus assay.*

**Results:**

Compared to the positive control, the administration of powder significantly reduced the PCE/PCE + NCE (polychromatic erythrocytes, normochromatic erythrocytes) ratio in treated mice. A significant increase in the percentage of MnPCE (micronucleus in polychromatic erythrocytes) in cyclophosphamide-treated bone marrow cells was observed. Compared with the positive controls, the groups treated with different doses in combination with cyclophosphamide presented a significant (p<0.0001) decrease in MnPCE in a dose-dependent manner. Compared to the positive control, PoSP significantly decreased MDA (malondialdehyde) levels in the livers of treated animals. The same things were observed in the kidneys and spleen. The catalase activity was also significantly increased in tissues, compared to negative control.

**Conclusions:**

These findings suggest that PoSP does not cause DNA damage and can prevent genotoxicity, probably through its antioxidant activities.

**Graphical abstract:**

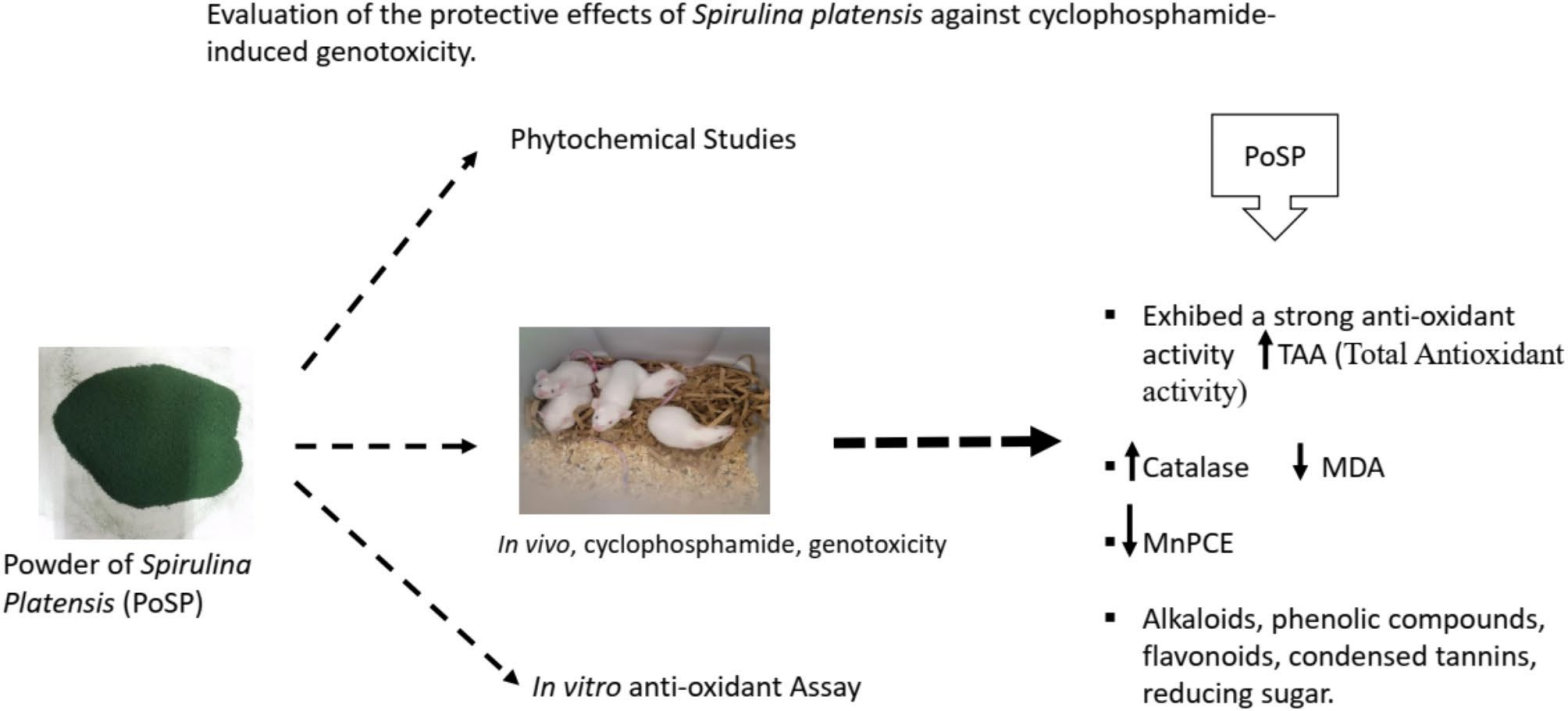

## Background

DNA undergoes multiple attacks from different sources, leading to the development of neoplastic diseases, including cancer [1, 2]. Those diseases require the systematic use of antineoplastic agents. Fast-growing cells are destroyed by these agents and caused the destruction of neoplastic tissue [3]. These agents can be toxic and cause irreversible tissue damage due to their narrow therapeutic range, which can lead to further complications. The growing concern about the effect of these agents, whether of food or drug origin, on human and animal health makes it essential to monitor their mutagenic potential to preserve the genetic heritage [4]. The exploration natural compounds from plants, has become essential, due to their chemopreventive, chemotherapeutic, and chemosensitising properties [5, 6].

Medicinal plants have been used for centuries to treat various diseases and are therefore an essential resource the manufacture of new pharmaceutical drugs. Scientific evidence has shown the benefits of using medicinal plants and active compounds from plants as safe and, non-toxic remedies [7]. Algae represent a wide variety of plants, rich in active metabolites and providing new ingredients for functional foods. They constitute an alternative source of dietary fibre, protein and minerals and are also considered as a source of bioactive compounds due to their content of various secondary metabolites which endow them with a multitude of biological activities such as antimicrobial, anti-inflammatory, antiviral and antitumour activities [8]. Numerous studies have shown that antioxidants can manage the undesirable side effects of antineoplastic drugs [3]. Similarly, epidemiological research had shown a correlation between the consumption of foods containing antioxidants and the prevention of various diseases [9].

The aim of the present study was to investigate PoSP, a blue-green alga of the Phormidiaceae family that is often used because of its rich nutritional constituents. Valuable proteins, essential amino acids, vitamins, β-carotene, minerals, essential fatty acids, polysaccharides and glycolipids are present in this alga [10]. Many studies had reported biological properties, such as antioxidant, anti-inflammatory, antineoplastic, lipid-lowering, antiviral, immunomodulatory, antimicrobial, anti-atherogenic, anti-diabetic and radioprotective effects [11]. Spirulina is non-toxic, with an LD50 of 2000 mg/kg [12]. Although spirulina is widely used, there is little data about to its genetic toxicity aspect. Based on its pharmacological properties, particularly its antioxidant properties, it is possible that spirulina has an anti-genotoxic effect [13]. The aim of this study was to evaluate the impact of PoSP on bone marrow micronuclei in mice. Specifically, the objective was to analyse the effects of PoSP on the genetic toxicity caused by cyclophosphamide, to assess the antioxidant properties in vitro and to identify the main phytochemical groups present in the powder.

## Methods

### Plant material

The plant material used was a hot-dehydrated PoSP produced in Benin. It comes from the production unit of the Institut Régional pour le Développement et la Santé, which is based in Benin. The powder was identified at the Department of Botany and Plant Ecology at the Faculty of Science, University of Lomé (Togo) and the specimen was deposited at the TOGOENSE herbarium under the number TOGO16001.

### Animal material

Male ICR mice, 5–6 weeks olds, weighing 25 ± 5 g, were provided by the animal facility of Department of Animal Physiology, University of Lomé - TOGO. Female mice were excluded because of hormone imbalance during the estrous cycle. The mice were housed in polypropylene cages and maintained under optimal conditions of temperature (25 ± 3 °C), relative humidity, and 12 h of light/ dark cycle. They were fed with a standard pellet diet with water *ad libitum*.

### Chemicals and reagents

Cyclophosphamide was purchased from Sigma‒Aldrich (St. Louis, MO). Commercial reagents such as May Grunwald, formaldehyde, and Giemsa were obtained from Biolabo SA (Paris, France).

### Phytochemical analysis of *Spirulina platensis* powder

#### Phytochemical screening

The screening was performed to determine qualitatively the presence of some phytochemical components, by using the standard methods described by Harbone and Trease [14].

#### Polysaccharides content

The method of Dubois [15] was used to determine the polysaccharides content. Polysaccharides give a yellow-orange colour in the presence of phenol and sulphuric acid. This is a sensitive reaction with stable coloration.

A volume of 200 μL of a 5% aqueous phenol solution and 1 mL sulphuric were added to 200 μL of the PoSP. The mixture was homogenized, incubated at 100 °C for 10 min. After incubation, the preparation was cooled for 30 min, protected from the light. Glucose at different concentrations (0–200 μg.ml^− 1^) served as standard and distilled water as blank. The absorbance was measured at 480 nm using US/VIS Spectrophotometer Wavelength.

#### Flavonoids content

According to the method of Motto et al. [3], flavonoids form a flavonoid-aluminium complex with aluminium chloride, which absorbs at 415 nm.

One mL of 2% aluminium chloride was added to 1 mL of the PoSP dissolved in ethanol. The mixture was incubated at laboratory temperature (25 ± 2 °C) for 10 min. After incubation, absorbance was measured at 415 nm against the blank. Ethanol served as the blank. Rutin (0–200 μg/mL) was used as a standard. Test triplicates were used (*N* = 3) and total flavonoids were expressed as mg Rutin equivalents/g spirulina powder.

### Determination of total phenols and tannins

The total phenolic compound and tannin contents of the PoSP were determined via the Ciocalteu method described by Maksimović et al. and Naczk and Shahidi [16, 17] was used to determine of the total phenolic and tannins contents. The phenolic compounds in the PoSP were oxidized by the Folin–Ciocalteu reagent which is reduced during the oxidation of the phenols to a mixture of blue oxides of tungsten and molybdenum.

To 200 μL of the solution of the PoSP, 200 μL of Folin-Ciocalteu reagent was added. After 30 min of incubation at room temperature, 800 μL of 700 Mm sodium carbonate was added to the mixture. The absorbance was read at 735 nm against a blank after a second phase of incubation for 120 min. Gallic acid at different concentrations served as standard. All tests were performed in triplicate.

Polyvinylpyrrolidone (PVPP) served to fix the tannins. It consisted of adding 500 μL of the dissolved PoSP to 10 mg of PVPP. After 30 min of incubation on ice twice, the mixture was homogenized and centrifuged. 200μL of the supernatants were added to Folin - Ciocalteu and sodium carbonate solutions as described previously. The absorbance was measured at 735 nm against a blank with *N* = 3.

Tannins were subtracted from total phenols based on the difference in OD.

OD tannins = OD powder– OD powder + PVPP

### In vitro antioxidant activity of *Spirulina platensis* powder

#### Total antioxidant capacity (TAC)

TAC is based on the reduction of molybdenum present as MoO_2_ + molybdate ions in the presence of an antioxidant to form a phosphate complex in an acidic medium [18].

3 mL of reagent solution (0.6 M sulfuric acid, 28 mM sodium phosphate and 4 mM ammonium molybdate) was added to 0.3 mL of the solution of the PoSP. The mixture was incubated at 95 °C for 90 min. Ascorbic acid served as standard. After cooling to room temperature, the absorbance of the solution was measured at 695 nm against a blank (methanol). The antioxidant activity was expressed as mg of ascorbic acid equivalent per g powder.

### Determination of the reducing power of *Spirulina platensis* powder

According to the methods of Oyaizu [19], reducing power is based on reducing ferric iron (Fe_3_+) to the iron salt (Fe_2_+) via antioxidants.

Different concentrations of the PoSP dissolved in methanol (0.5 ml) were mixed with (2.5 ml) of phosphate Buffer (pH = 6.6) and potassium ferricyanide at 1% (2.5 ml). This mixture was kept at 50 ºC in water bath for 20 min. After cooling, 2.5 ml of 10% trichloroacetic acid was added to stop the reaction. After 10 min of centrifugation at 3000 rpm, 2.5 mL of the supernatant was collected, and 2.5 mL of distilled water and 0.5 mL of a 1% ferric chloride (FeCl_3_) solution were added. The absorbance of the reaction was measured at 700 nm against a blank (methanol). The absorbance was measured at 700 nm.

Reducing power intensity (%) = [(OD test-OD blank)/OD blank]x 100

### Evaluation of antigenotoxic properties of *Spirulina platensis* powder

Micronuclei formation in erythrocytes is due to chromosomal damage induced by chemicals during mitotic division [20]. The increased frequency of micronuclei in treated animals indicates structural chromosomal damage [21].

### Experimental protocol

This test was carried out according to OECD LD 474 recommendations for mice. A total of 25 ICR mice weighing (25 ± 5 g) were divided into 5 groups of 5 animals. Three different concentrations of the PoSP were tested to assess the prevention of genotoxicity and cytotoxicity in vivo in mouse bone marrow cells. The choice of the three concentrations was based on previous work carried out by Kougblenou et al. [22].

Three doses of 37.5 mg/kg, 75 mg/kg, and 150 mg/kg of PoSP dissolved in distilled water had been administered daily for 7 days per os:

Group 1: Negative control, was treated with distilled water;

Group 2: Positive control, was treated with distilled water

Group 3: treated with PoSP at 37.5 mg/kg

Group 4: treated with PoSP at 75 mg/kg

Group 5: treated with PoSP at 150 mg/kg

After 7 days of pretreatment, all groups of mice received cyclophosphamide at a dose of 100 mg/kg (prepared in 0.9% NaCl) via intraperitoneal route, except for group 1, which received NaCl (0.9%). Thirty (30) hours after injection, the animals were anesthetized using light diethyl ether. Blood samples were collected via retro-orbital sinus into EDTA tubes for complete blood count. Then, animals were euthanized by cervical dislocation and the bone marrow, liver, spleen, and kidneys were quickly removed.

All the mice were observed daily for sign of toxicity during the treatment period. The body weight of each mouse was recorded twice before the administration of spirulina powder and euthanasia.

### Preparation slides and staining

- Preparation of slides.

Smears were prepared according to the methods of Krishna et al. [23]

After cervical dislocation, the femurs of the mice were removed and cleaned in saline solution. The epiphyses were then cut, and the bone marrow was washed and recreated with 0.9% NaCl in tubes. The pellet was recovered after 7 min of centrifugation at 1000 × g at room temperature. Formaldehyde 4% was added to the pellet to preserve cell cytoplasms. Thus, the pellets were spread on slides, and dried in open air. The smears were stained with the May Grunwald for 2 min, and Giemsa 5% for 30 min.

- Microscopic observation of stained slides.

The stained smears were methodically read using a tinocular light microscope (Olympus), in magnification 1000 (10 × 100). The images have been recorded directly on a laptop connected to the PC Ocular camera type 049002-VGA (Germany) integrated under a microscope.

### Counting of micronucleus and erythrocytes

For each animal, the proportion of polychromatic erythrocytes (%PCE) (also known as immature erythrocytes) in the total number of erythrocytes (polychromatic + normochromatic) was determined by counting 1000 erythrocytes to confirm the presence or absence of cytotoxicity. Similarly, the incidence of micronucleated polychromatic erythrocytes (%MnPCE) was determined on the basis of the examination of 5000 polychromatic erythrocytes per animal according to the formulas below:

%MnPCE = (Number MnPCE)/(Number PCE + NCE))x 100

% PCE = (Number PCE)/(Number PCE + Number NCE)) × 100

MnPCE = Micronucleus in polychromatic erythrocytes

PCE = Polychromatic erythrocyte

NCE = Normochromatic erythrocyte

### Complete blood count

Blood counts were performed on whole blood collected in EDTA tubes. The number of red blood cells (RBCs), white blood cells (WBCs), platelets, hematocrit (Ht), mean corpuscular hemoglobin concentration (MCHC), and hemoglobin (LH) level were determined using URIT-5160 hematology analyzer and URIT Medical Electronic Co., Ltd reagents.

### Determination of oxidative stress markers

#### Catalase content in the liver, spleen, and kidney

Through optimized enzymatic combination, catalase activity has been measured in spectrophotometer by the formation of the yellow and stable complex with hydrogen peroxide and ammonium molybdate [24]. Each previously frozen organ was carefully rinsed with 9‰ ice-cold NaCl and then ground in 1.0 mL of sodium‒potassium phosphate buffer. To 100 μL of homogenate, 500 μL of substrate was added, followed by incubation at 37 °C for 60 s. The reaction was stopped by adding 500 μL of 32.4 mM/L ammonium molybdate. The absorbance of the yellow color resulting from the formation of the molybdate-H_2_O_2_ complex was measured at 405 nm.

Catalase activity

kU/L) = ((A (sample)-A (sample blank))/(A (substrate blank)-A (buffer blank)) × 271

Sample blank = 100 μL sample + 500 μL buffer + 500 μL ammonium molybdate

Substrate blank = 100 μL of buffer + 500 μL of substrate + 500 μL of ammonium molybdate

Buffer blank = 100 μL of buffer + 500 μL of buffer + 500 μL of ammonium molybdate

The catalase unit breaks down 1 μM hydrogen peroxide/1 min in this method.

Substrate = 65 μM/mL hydrogen peroxide in 60 mM/L sodium‒potassium phosphate buffer.

#### Dosages of malondialdehyde in the liver, spleen, and kidney

Lipid peroxidation in the liver, kidney, and spleen was assessed by quantifying malondialdehyde (MDA), a metabolic product resulting from free radical attack on membrane lipids (polyunsaturated fatty acids) [25].

1-methyl-2-phenylindole at 10 nM was activated by mixing it with a 32 μM FeCl3 75% of 1-methyl-2-phenylindole in acetonitrile + 25% FeCl3 in methanol).

Organs were washed in 9 g/L NaCl and ground in 150 mM Tris HCl buffer. The reaction medium consisted of 650 μL of activated 1-methyl-2-phenylindole, 250 μL of homogenate of liver, spleen, or kidney, 150 μL of 12 N HCl; and 10 μL of 0.1 M butyraled hydroxy toluene (BHT) solution.

### Statistical analysis

The data are expressed as the mean ± SEM (standard error of the mean) and were analyzed using Graph Pad Prism 7.0 software. Analysis of variance (ANOVA) followed by Bonferroni correction was used to measure significant differences between groups with a significance level of <0.05.

## Results

### Phytochemical screening

Phytochemical screening of the PoSP revealed as the presence of alkaloids and phenolic compounds, including flavonoids, condensed tannins, triterpenes, reducing compounds, and reducing sugars.

### Total phenol and tannin, polysaccharide, and total flavonoid contents

The results revealed that PoSP contains a significant amount of total phenols, tannins, flavonoids and polysaccharides. Their contents are summarized in Table 1.

**Table 1:** Concentrations of total phenols and tannins, total flavonoids, and polysaccharides in the PoSP

Total phenols mg Eq AG/g	Tannins mg Eq AG/g	Flavonoids mg Eq *R*/g	Polysaccharides Eq Gluc/g
6.81 ± 0.20	1.36 ± 0.29	129.75 ± 2.87	93.65 ± 0.07

mg Eq. AG/g: The concentrations of total phenols and tannins contained in the PoSP were expressed in mg of gallic acid equivalent/g of *Spirulina platensis* powder; (mg Eq. R/g): those of total flavonoids were expressed in mg of rutin equivalence/g of *Spirulina platensis* powder; (Eq. Gluc/g): those of the polysaccharides were expressed as the equivalent of glucose/g of spirulina powder. ANOVA followed by Bonferroni correction was used to measure significant differences between groups with a significance level of <0.05

### In vitro antioxidant activity of *Spirulina platensis* powder

#### Total antioxidant capacity

The total antioxidant capacity of ascorbic acid increases proportionally with concentration. The antioxidant activity is expressed in mg of ascorbic acid equivalent per g of PoSP and is 47.75 ± 1.10 mg Eq AA/g. This value represents the proportion of all the antioxidant compounds present in the PoSP that act in both the hydrophilic and lipophilic compartments.

#### Reducing power

**Fig. 1:**
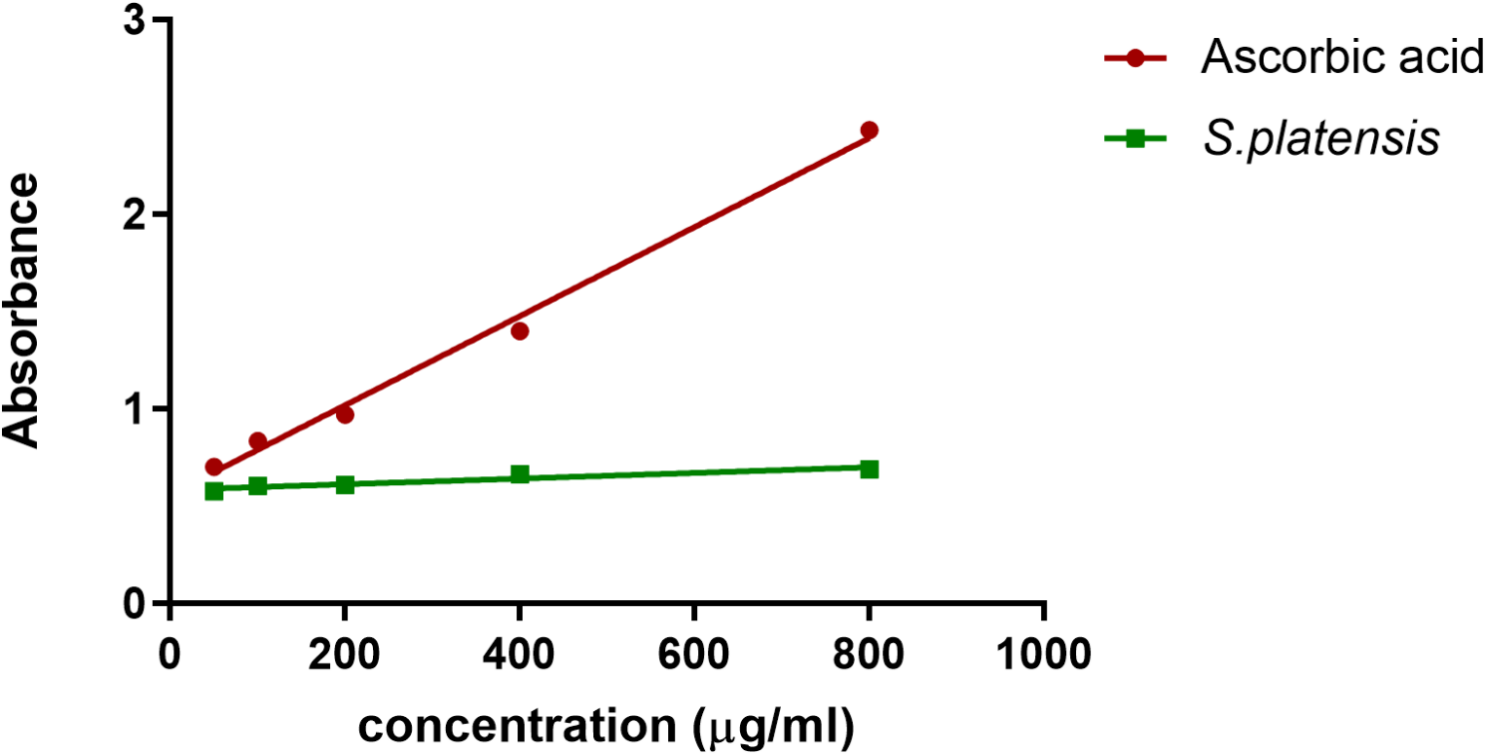
Reducing power of *Spirulina* powder

Figure 1 reveals that PoSP reduces iron in a dose-dependent manner. Compared with the reducing power of the reference (ascorbic acid), the reducing power of the PoSP was lower and constant regardless of the concentration.

### Anti-genotoxic activity of *Spirulina platensis* powder

#### Effects of *Spirulina platensis* powder on mouse weight and organ weight

The animals behaved normally during the experiment. No significant difference in weight gain was observed between the treated animals and the negative control animals (Table 2). Observation of the organs revealed normality, with no signs of atrophy or hypertrophy (Table 3).

**Table 2:** Changes in weight (in grams) at the beginning and end of the experiment

Batches	weight of mice (g)						
	1	2	3	4	5	MEAN ± SEM	Weigth gain (g)
NC							
Start End	21 25	23 24	28 30	24 24	24 25	24.00 ± 2.55 25.60 ± 2.51	1.60 ± 0.98
PC							
Start End	28 29	29 30	28 31	29 28	27 26	28.20 ± 0.37 28.80 ± 0.86	0.60 ± 0.75
SP 37.5							
Start End	30 31	22 25	30 30	29 32	23 22	26.80 ± 1.77 28.00 ± 1.92	1.60 ± 0.80
SP 75							
Start End	27 29	30 30	27 31	28 30	24 23	27.20 ± 0.97 28.60 ± 1.44	1.40 ± 0.87
SP 150							
Start End	32 34	29 30	25 27	23 25	25 25	26.80 ± 1.63 28.20 ± 1.72	1.40 ± 0.40

NC: normal control; PC: positive control; SP 37.5: mice treated with 37.5 mg/kg *Spirulina* powder; SP 75: mice treated with 75 mg/kg *Spirulina* powder; SP150: mice treated with 150 mg/kg *Spirulina* powder

**Table 3:** Relative weights of the liver, kidney, and spleen

	NC	PC	SP 37.5	SP 75	SP 150
Liver	5.03 ± 0.21	4.95 ± 0.30	4.82 ± 0.36	4.70 ± 0.07	4.74 ± 0.08
Kidneys	1.36 ± 0.15	1.35 ± 0.07	1.31 ± 0.11	1.33 ± 0.07	1.36 ± 0.05
Spleen	0.28 ± 0.08	0.21 ± 0.02	0.24 ± 0.04	0.27 ± 0.06	0.28 ± 0.02

NC: normal control; PC: positive control; SP 37.5: mice treated with 37.5 mg/kg *Spirulina* powder; SP 75: mice treated with 75 mg/kg *Spirulina* powder; SP150: mice treated with 150 mg/kg *Spirulina* powder. ANOVA followed by Bonferroni correction was used to measure significant differences between groups with a significance level of <0.05

#### Effects of *Spirulina platensis* powder on hematological parameters

Compared to the normal control, the red blood cell count significantly (p<0.05) increased at SP 150. Compared to of the negative control, the white blood cell count significantly (p<0.01) decreased in the positive control. Compared to the negative control, the platelet count of the positive control significantly (p<0.05) decreased. Compared to the positive control, the Hb levels and Hct significantly increased in a dose-dependent manner, as indicated in Table 4.

**Table 4:** Effects of *Spirulina platensis* powder on hematological parameters

	NC	PC	SP 37.5	SP 75	SP 150
RBC(10^− 6^ /μL)	7.14 ± 0.02	6.14 ± 0.04^#^	7.16 ± 0.25^*^	8.04 ± 0.17^**^	8.19 ± 0.04^****^
HL (g/dL)	14.76 ± 0.24	13.44 ± 0.25	14.74 ± 0.18	15.78 ± 0.24^**^	15.50 ± 0.17^**^
Hte (%)	43.46 ± 2.42	38.06 ± 0.56	44.82 ± 1.95^*^	45.94 ± 1.15^**^	45.94 ± 1.15^**^
MGV (fl.)	64.34 ± 1.66	62.28 ± 1.11	61.78 ± 1.14	60.59 ± 1.12	61.09 ± 2.13
MCHR (pg)	21.02 ± 0.36	22.36 ± 0.39	20.34 ± 0.52	19.70 ± 0.32	19.38 ± 0.32
MCHC(g/dL)	32.94 ± 1.16	36.00 ± 0.87	33.00 ± 0.99	33.32 ± 0.96	31.90 ± 1.36
WBC(10^3^/mL)	2.62 ± 0.04	1.28 ± 0.09^##^	1.42 ± 0.16	1.64 ± 0.14	1.61 ± 1.36
PLT(10^3^/μL)	513.80 ± 59.76	185.40 ± 12.26^#^	339.80 ± 34.52^***^	489.60 ± 25.76^***^	596.60 ± 50.40^***^

WBC: white blood cells; HL: hemoglobin level; Hte: hematocrit; MGV: mean globular volume; MCHR: mean corpuscular hemoglobin level; MCHC: mean corpuscular hemoglobin concentration; RBC: red blood cell; PLT: platelet. The results were analyzed with one-way ANOVA, and the data are presented as the means ± SEM. # *p* < 0.05 ##p<0.01 (compared with the normal control); *p < 0.05 (compared with the positive control). **p<0.01 (compared with the positive control); ***p<0.001 (compared with the positive control); ****p<0.0001 (compared with the positive control). NC: normal control; PC: positive control; SP 37.5: mice treated with 37.5 mg/kg *Spirulina* powder; SP 75: mice treated with 75 mg/kg *Spirulina* powder; SP150: mice treated with 150 mg/kg *Spirulina* powder. WBC: white blood cells; HL: hemoglobin level; Hte: hematocrit; MGV: mean globular volume; MCHR: mean corpuscular hemoglobin level; MCHC: mean corpuscular hemoglobin concentration; RBC: red blood cell; PLT: platelet. ANOVA followed by Bonferroni correction was used to measure significant differences between groups with a significance level of <0.05. # *p* < 0.05 ##p<0.01 (compared to the normal control); *p < 0.05 (compared to the positive control). **p<0.01 (compared with the positive control); ***p<0.001 (compared to the positive control); ****p<0.0001 (compared to the positive control)

#### Effects of *Spirulina platensis* powder on micronucleus levels in bone marrow cells

**Fig. 2:**
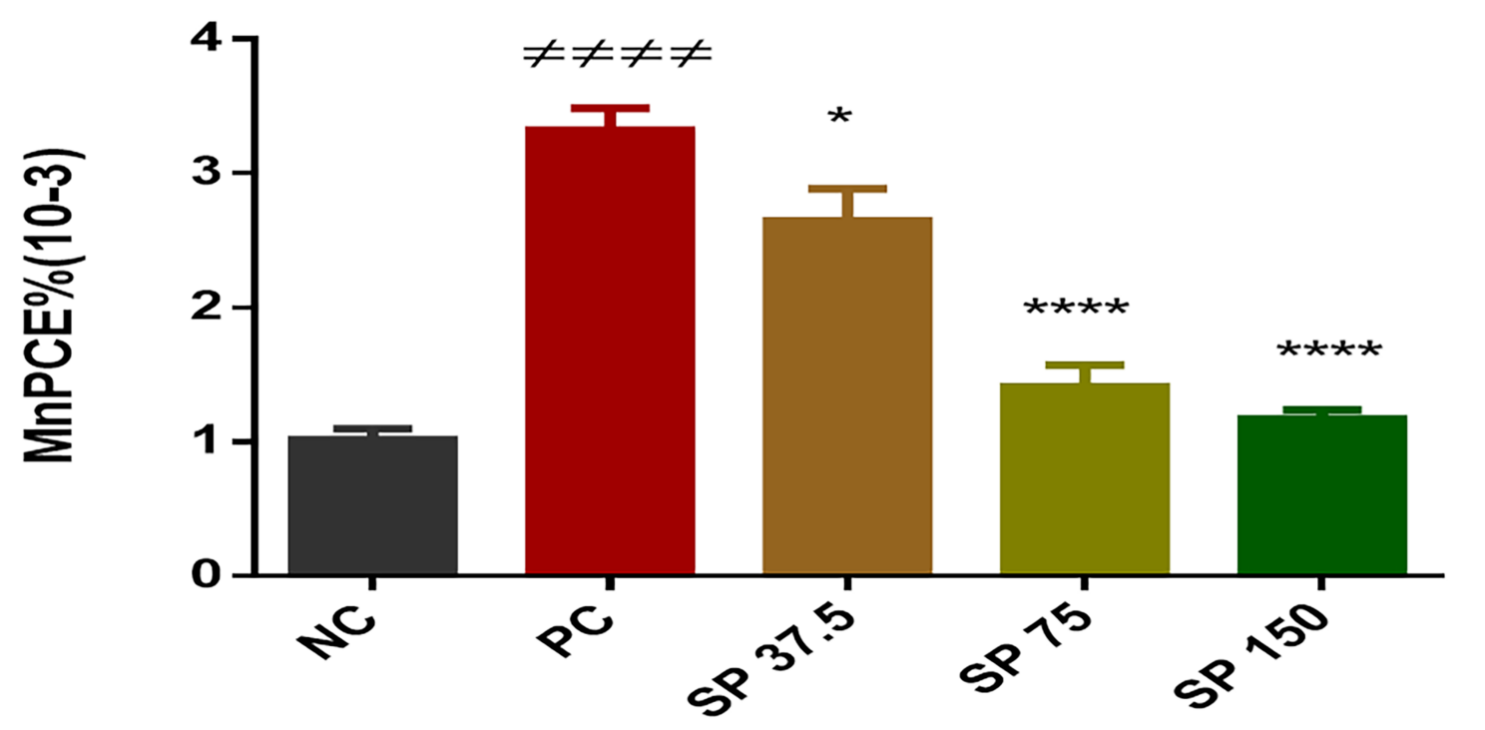
Percentage of micronuclei in polychromatic erythrocytes. NC: normal control; PC: positive control; SP 37.5: mice treated with 37.5 mg/kg *Spirulina* powder; SP 75: mice treated with 75 mg/kg *Spirulina* powder; SP150: mice treated with 150 mg/kg *Spirulina* powder. # # # # *p* < 0.0001 (compared to the normal control); *p<0.05 (compared to the positive control); ****p < 0.0001 (compared to the positive control)

Comparedto the negative control group, the groups pretreated with PoSP (37.5, 75, or 150 mg/kg) followed by cyclophosphamide presented a significant (*p<0.05*,* p<0.0001*) dose‒dependent reduction in the percentage of MnPCE. In contrast, the positive control, which received only cyclophosphamide, had the highest percentage of MnPCE, as indicated in Fig. 2.

**Fig. 3:**
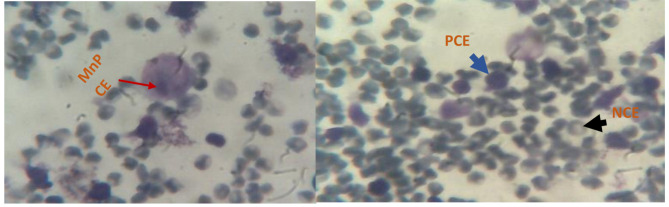
Photos of micronucleated polychromatic erythrocytes (MnPCEs), polychromatic erythrocytes (PCEs) and normochromatic erythrocytes (NCE) in mouse bone marrow cells

Figure 3 shows micronucleated polychromatic erythrocytes (MnPCEs), polychromatic erythrocytes (PCEs), and normochromatic erythrocytes (NCE).

The stained smears were methodically read using a tinocular light microscope in magnification 1000 (10 × 100).

#### Action of *Spirulina platensis* powder on bone marrow cells following cyclophosphamide administration

Administration of the PoSP at the indicated doses did not significantly increase the ratio of polychromatic to total erythrocytes (PCE%) compared to normal control. A significant increase (p<0.05) was observed in the positive control compared to the normal control. The frequency of MnPCEs was significantly greater (# # # # *p* < 0.0001) in cyclophosphamide-treated animals than in NaCl-only animals, as indicated in Table 5.

**Table 5:** Effects of *Spirulina platensis* powder followed by cyclophosphamide administration on bone marrow cells

Mice	Normal Control		Positive Control		SP 37.5 + C		SP 75 + C		SP 150 + C	
	MnPCEs/5000 PCEs	PCEs%	MnPCEs/5000 PCEs	PCEs%	MnPCEs/5000 PCEs	PCEs%	MnPCEs/5000 PCEs	PCEs%	MnPCEs/5000 PCEs	PCEs%
1	5	51.64	17	43.14	13	44.72	8	43.73	5	41.87
2	6	46.42	9	43.08	15	46.80	6	45.96	6	45.94
3	5	56.61	17	39.77	15	42.47	10	45.52	6	45.08
4	5	46.43	19	41.77	14	44.93	6	42.24	7	42.42
5	4	41.04	15	44.43	9	47.39	5	45.03	5	45.63
**M ± SEM**	**5.00 ± 0.32**	**48.43 ± 3.10**	**15.40 ± 1.72** ^**####**^	**42.44 ± 0.79** ^**#**^	**13.22 ± 1.11**	**45.26 ± 0.87**	**7.00 ± 0.89** ^***^	**44.50 ± 1.41**	**5.80 ± 0.37** ^****^	**44.19 ± 0.85**

Five batches of 5 mice were formed. The normal control was treated with distilled water; the positive control was given cyclophosphamide at 100 mg/kg; SP 37.5 + C; SP 75 + C; and SP 150 + C were pretreated with total extract at doses of 37.5, 75, and 150 mg/kg and subsequently given cyclophosphamide at 100 mg/kg. # *p* < 0.05 # # # # *p* < 0.0001 (compared to the normal control); ***p < 0.001 ****p < 0.0001 (compared to the positive control)

PCE = micronucleus in polychromatic erythrocytes; PCE = polychromatic erythrocytes; NCE = normochromatic erythrocytes

### Effects of *Spirulina platensis* powder on two oxidative stress parameters

#### MDA assay

**Fig. 4:**
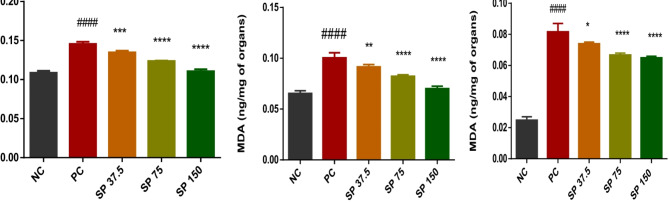
Malondialdehyde levels in the kidneys, liver and spleen. NC: normal control; PC: positive control; SP 37.5: a group of mice treated with 37.5 mg/kg Spirulina powder; SP 75: a group of mice treated with 75 mg/kg Spirulina powder; SP150: a group of mice treated with 150 mg/kg Spirulina powder. ANOVA followed by Bonferroni correction was used to measure significant differences between groups with a significance level of <0.05. # # # # *p* < 0.0001 (compared to the normal control); ****p < 0.0001 (compared with to the positive control)

The pre- and postcyclophosphamide treatment groups presented significant (p<0.0001) dose‒dependent reductions in the levels of MDA in the spleen, kidney, and liver. The positive control that received only cyclophosphamide had the highest MDA levels in the spleen, kidney, and liver, as indicated in Fig. 4.

#### Catalase assay

**Fig. 5:**
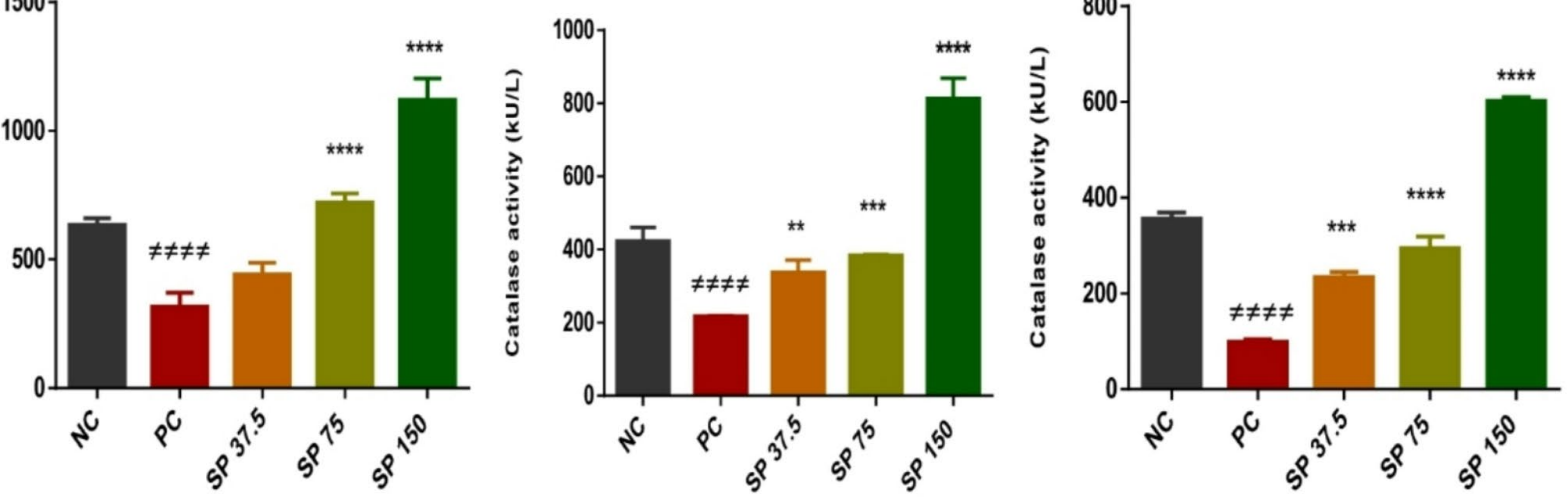
Catalase levels in the kidney, liver, and spleen. NC: normal control; PC: positive control; SP 37.5: a group of mice treated with 37.5 mg/kg *Spirulina* powder; SP 75: a group of mice treated with 75 mg/kg *Spirulina* powder; SP150: a group of mice treated with 150 mg/kg *Spirulina* powder. # # # # *p* < 0.0001 (compared with the normal control); **p<0.01; ***p<0.001; ****p < 0.0001 (compared with the positive control). NC: normal control; PC: positive control; SP 37.5: mice treated with 37.5 mg/kg *Spirulina* powder; SP 75: mice treated with 75 mg/kg *Spirulina* powder; SP150: mice treated with 150 mg/kg *Spirulina* powder. ANOVA followed by Bonferroni correction was used to measure significant differences between groups with a significance level of <0.05 # # # # *p* < 0.0001 (compared to the normal control); *p<0.05 (compared to the positive control); ****p < 0.0001 (compared to the positive control)

The pretreated groups presented significant dose‒dependent increases in catalase activity in the spleen, kidney, and liver. The cyclophosphamide-only positive control had the lowest catalase activity in the spleen, kidney, and liver, as indicated in Fig. 5.

## Discussion

This study allowed to evaluate, the genotoxicity and cytotoxicity of *Spirulina platensis* powder.

Micronuclei are present in the bone marrow erythroblasts or peripheral blood cells of mice. They are extranuclear bodies containing damaged and/or whole chromosome fragments that have not been incorporated into the nucleus after cell division [21]. DNA breakage, chromosomal aberrations, mitotic apparatus dysfunction, and interference with DNA synthesis are possible explanations for micronucleus formation [3]. The in vivo micronucleus test is one of the genotoxicity tests recommended by international regulatory agencies and government institutions for the evaluation of new substances. Before and after cyclophosphamide administration, no abnormalities in the general behavior of the animals were observed. The weight gain of the mice before necropsy was not significantly different (*p* ≥ 0.05) from that of the normal controls. The organs showed no signs of atrophy or hypertrophy after necropsy. The chemotherapeutic utility of alkylating agents involves their ability to form diverse DNA adducts that suitably alter DNA structure or function or both in an attempt to have a cytotoxic effect on cells. Many of them undergo a very complex activation process before being able to generate reactive intermediates via the microsomal oxidation system in the liver, which produces 4-hydroxycyclophosphamide, which is a cytotoxic metabolite known to cause myelosuppression [26]. Cyclophosphamide causes DNA cross-linking and the inhibition of DNA synthesis by acting on both cyclic and intermitotic cells, resulting in a general deficiency of the cells that make up the immune system [27]. In our study, there was a significant decrease (p<0.001) in WBCs in the positive control batch compared with those in the normal control batch. The difference in WBCs was not significant in batches reciving different doses of PoSP were compared to the positive control. WBCs are among the main components of the immune system and are produced in the bone marrow. A low level is often linked to a problem with the machinery making enough WBC from the bone marrow. These findings suggest that PoSP is not cytotoxic and may act as a modulator of WBC numbers. A significant decrease (p<0.05) in thrombocytes and red blood cells (RBCs) was revealed in the positive control batch compared to the negative control batch, batches of mice pretreated with PoSP showed a dose-dependent reduction. Thrombocytes are crucial mediators of hemostatic functions and play a key role in inflammatory process [28]. Erythrocytes play a role in the immune system and have the main function of exchanging respiratory gases. PoSP therefore has a positive effect on hemostasis, respiratory gas exchange, and the inflammatory process. These results are consistent with those of El-Naggar et al. [29], who demonstrated that the microalga *Spirulina platensis* ameliorated cyclophosphamide-induced hematological, hepatic, and renal toxicity in male albino mice. The number of micronucleated polychromatic erythrocytes (MnPCE) in the bone marrow of mice corresponds to the mutagenic potential of cyclophosphamide-induced chromosomal damage [23]. A significant increase in MnPCE levels in animals treated with a test molecule is indicatives cyclophosphamide-induced chromosomal damage [21]. PoSP administered at different doses caused a significant decrease in the frequency of MnPCE in the bone marrow cells of the mice compared to the positive control animals, which had the highest percentage of MnPCE. Our results are comparable to those of Motto et al. [3], who evaluated the genotoxicity and protective effect of *Anogeissus leiocarpus* roots against cyclophosphamide-induced DNA damage in vivo in ICR mice. One of the derivatives of cyclophosphamideis acroleinwhichinduces oxidative stress, then DNA damage in normal cells and toxicity to various target organs. It activates reactive oxygen species and nitric oxide production, leading to the formation of peroxynitrite, which damages lipids, proteins, and DNA inside cells [30]. The presence or absence of cytotoxicity was assessed by measuring the PCE/NCE ratio in 5000 erythrocytes (PCE + NCE) of polychromatic erythrocytes (EPC). Bone marrow cell proliferation is affected by the presence of a toxic substance when the PCE/NCE ratio decreases [23]. Compared to the negative control, the administration of PoSP did not significantly decrease the PCE/NCE + NCE ratio in treated mice. The cytotoxic activity is therefore not attributed to PoSP. In constrast, the powder protects against cyclophosphamide-induced genotoxicity in the bone marrow in a dose-dependent manner. Compared to of the positive control, the incidence of MnPCE was 1.72.10^− 3^ versus 3.320.10^− 3^ at 150 mg/kg. These results are comparable to those of Thybaud et al. [21], who demonstrated the chemoprotective effects of hesperidin against cyclophosphamide-induced genotoxicity in the bone marrow cells of male NMRI mice. The cytotoxic effect of cyclophosphamide is attributed to oxidative stress, inflammation, and apoptosis [31]. The use of compounds with antioxidant properties is a major asset in counteracting the effect of cyclophosphamide. MDA is a marker of membrane lipoperoxidation and increases in the organism after cyclophosphamide administration following the eventual destruction of membrane lipids with the formation and propagation of lipid radicals; the latter causes a rearrangement of the double bond in unsaturated lipids [32]. Compared tothe positive control, PoSPsignificantly reduced MDA levels in the livers of treated animals. The same findings were observed in the kidneys and spleen. Increased MDA levels not only reduce the levels of endogenous antioxidant defense markers such as SOD, CAT, and GSH. SOD normally converts superoxide radicals into H_2_O_2_, which dissociates into H_2_O and O_2_ in the presence of catalase [33]. The catalase activity in the liver, kidney, and spleen significantly increased compared to in the negative control, which explains the low MDA level in the mice fed spirulina powder.

Although the exact mechanism of the chemoprotective effect is unknown, free radical scavenging is responsible for the inhibitory effects of natural compounds on the clastogenic activity induced by genotoxic agents [34]. The main components of PoSP are alkaloids, phenolic compounds, triterpenes, and reducing compounds, as shown by some works of [22, 35], who investigated the effects of PoSP on metabolic syndrome in Sprague Dawley rats and the phytochemical screening and antioxidant activity of the seaweeds *Gracilaria corticata* and *Spirulina platensis*. The chemoprotective properties of algae are thought to be due to the presence of these compounds. Phenolic compounds, including ellagic acid, a natural polyphenol, have been evaluated for their anti-genotoxic effect and antioxidant activity against cyclophosphamide-induced renal stress and genotoxicity in Swiss albino mice [36]. Flavonoids exert a genoprotective effect by reducing oxidative damage to DNA through chelation of divalent cations; reducing lipoperoxidation, reactive nitrogen species and reactive oxygen species; and enhancing cellular defense systems (SOD, CAT, GSH, etc.). They also act by inhibiting the bioactivation of genotoxic agents through the inhibition of cytochrome P450 [37]. The chemoprotective effects of hesperidin, a flavonoid, were evaluated for its protection against genotoxicity against cyclophosphamide according to previous studies [38]. The antioxidant effects of saponosides, particularly triterpenes, protect against cyclophosphamide-induced damage to and apoptosis of bone marrow cells and peripheral blood lymphocytes in mice. Similarly, they reduce cyclophosphamide-induced genotoxicity and cytotoxicity in normal cells by increasing superoxide dismutase and glutathione and inhibiting the increased level of malondialdehyde caused by cyclophosphamide. They can also restore hematopoietic system function [39]. Numerous studies in the literature have demonstrated the chemoprotective capacity of polysaccharides in natural products. Indeed, polysaccharides increase the number of WBCs and nucleated cells in the blood and DNA in the bone marrow of mice. In vitro antioxidant activities have shown that PoSP can reduce metals and possesses high total antioxidant activity. This antioxidant activity is due to the phenolic compounds present in the algae [40].

Given our results, it is impossible to state with any certainty the exact mechanisms of action of the PoSP. Molecular studies by HPLC, Western blot, real-time RT‒PCR, determination of the effect of the powder on DNA denaturation, and histopathological examinations and analyses will be necessary.

## Conclusions

This study revealed that PoSP is not genotoxic but prevents cyclophosphamide-induced genotoxicity. The PoSP considerably reduces oxidative stress through the stimulation of antioxidant enzymes. These results support the idea that PoSP could be used to prevent the toxicity of chemotherapeutic drugs such as cyclophosphamide. However, further studies are needed to understand the exact mechanism of action of the powder. Molecular studies by HPLC, Western blot, real-time RT‒PCR, determination of the effect of the powder on DNA denaturation, and histopathological examinations and analyses are necessary.

## Data Availability

All the data analyzed during this study are included in this manuscript.

## Abbreviations

BHT: Butyraled Hydroxy Toluen
CAMES: Conseil Africain et Malgache Pour l’Enseigenment Supérieur
CAT: Catalase
DNA: Deoxyribonucleic acid
EDTA: Ethylene Diamine Tetraacetic Acid
Eq. Gluc/g): those of the polysaccharides were expressed as the equivalent of glucose/g of *Spirulina* powder
Fe^2+^: Ferrous ion
Fe^3+^: Ferric ion
FeCl_3_: Ferric Chloride
GSH: Reduced L-Glutathion
H_2_O: Water Molecule
H_2_O_2_: Hydrogen peroxide
HCl: Hydrochloric acid
Ht: Hematocrit
LD_50_: Lethal Dose
LH: Hemoglobin Level
MCHC: Mean Corpuscular Hemoglobin Concentration
MCHR: Mean Corpuscular Hemoglobin Level
MDA: Malondialdehyde
mg Eq AG/g: The concentrations of total phenols and tannins contained in the powder were expressed in mg of gallic acid equivalent/g of *Spirulina platensis* powder
mg Eq. R/g: Those of total flavonoids were expressed in mg of rutin equivalence/g of *Spirulina platensis* powder
MGV: Mean Globular Volume
MnPCE: Micronucleus in Polychromatic Erythrocytes
NaCl: Sodium Chloride
NCE: Normochromatic Erythrocytes
O_2_: Oxygen
OECD: Organization for Economic Co-operation and Devevelopment
PCE: Polychromatic Erythrocytes
PLT: Platelet
PoSP: *Spirulina platensis* powder
PVPP: Polyvinylpolypyrrolidone
RBC: Red Blood Cell
SOD: Superoxide Dismutase
SP: *Spirulina platensis*
TAATAC: Total Antioxidant activityCapacity
WBC: White blood cells
